# Functional and Idiopathic Cervical Dystonia in Two Family Members: A Challenging Diagnosis

**DOI:** 10.5334/tohm.558

**Published:** 2020-12-11

**Authors:** Elena Antelmi, Enrico Conti, Myriam Carecchio, Michele Tinazzi

**Affiliations:** 1 Neurology Unit, Movement Disorders Division, Department of Neurosciences, Biomedicine and Movement Sciences, University of Verona, Verona, IT; 2 Department of Neuroscience, University of Padua, Padua, IT

**Keywords:** cervical dystonia, familial dystonia, functional movement disorder, endophenotypic trait

## Abstract

**Background:**

Cervical dystonia (CD) often occurs in the same family.

**Case report:**

A 40-year-old woman presented with a longstanding history of CD and signs of inconsistency at history taking and neurological examination; her 65-year-old mother was diagnosed instead with idiopathic CD, which had begun 7 years after the onset of CD in her daughter.

**Discussion:**

Idiopathic and functional CD share common clinical and endophenotypic traits, making the differential diagnosis particularly challenging. A complete examination is warranted.

## Introduction

Cervical dystonia (CD) often occurs in the same family, but known genes, frequently with an autosomal dominant pattern of inheritance and reduced penetrance, are responsible for less than 1% of cases [1]. Tremulous CD is more likely to be familial than non-tremulous CD [2]. Clinical diagnosis remains pivotal, but differential diagnosis between functional CD (fCD) and idiopathic CD (iCD) is challenging since both share common clinical [3,4,5,6,7] and neurophysiological traits [8,9,10,11,12]. Here we present the case of two family members in which differential diagnosis between fCD and iCD was made.

## Case-report

Case 1. A 40-year-old woman came to our observation because of a longstanding history of CD. History taking was remarkable for sudden onset of symptoms at age 26 years, with her neck turned to the left. At the time, a neurologist made a diagnosis of iCD. Pharmacological treatment (a 2-month trial with anticholinergic drugs and a single trial of botulinum toxin injection) failed. Two years later, at age 28 years, the symptoms spontaneously improved and remitted. After another 2 years, at age 30, CD recurred, with the neck turned to the right. Symptoms spontaneously improved over the following year and the patient stayed symptom-free for the next 8 years. CD then recurred 1 year before current presentation; pharmacological treatment comprising a 40-day course of therapy with an anticholinergic drug and a trial of botulinum toxin injection (Botox 45 units, U) into the left sterocleidomastoid muscle (SCM), 25 U into the right SCM, and 30 U into the right splenius capitis failed to relieve symptoms.

History taking disclosed that 7 years after symptom onset her mother, too, had developed CD symptoms. The patient did not complain of pain, while stress and anxiety were reported to worsen the torticollis. Neurological examination (Video 1) at current presentation revealed her neck turned to the right, with fluctuation of severity on turning the head. Neck turning worsened with attention and movement but disappeared with distraction maneuvers. Response to the sensory trick was absent. There was mild hypertrophy of the left SCM, some elevation of the right shoulder, and slight dystonic posturing of the right hand. Mild abasia during tandem gait was noted. Imaging studies (brain and cervical magnetic resonance) and laboratory tests for metabolic disorders were negative.

**Video 1:** **The daughter.** Segment 1 shows the 40-year-old daughter with her neck turned to the right and very mild posturing of the hands (mainly the right hand) while keeping her arms outstretched. The neck turning fluctuated in severity, worsening with attention and movement and while walking. The posture disappeared with distraction maneuvers and at times faded completely during the examination and/or when attention was distracted. Segment 2 at 2-months follow-up assessment and Segment 3 at 4-months follow-up assessment show the disappearance of symptoms and signs; although mild hypertrophy of the left SCM and slight right shoulder elevation can still be seen.

The inconsistency at the clinical history (acute symptom onset, long and recurrent periods of remission, head turning to the left and then to the right) and at clinical examination (positive response to distractive maneuvers, abasia), and lack of response to standard treatments, aroused suspicion of a possible functional overlay. A treatment decision was made. Psychological and physical rehabilitation brought progressive improvement of symptoms and complete remission within 1 month of the initiation of therapy. Follow-up assessment at 2 and 4 months after the end of treatment (Video 1 – second and third segments) confirmed the absence of symptoms.

Case 2: The patient’s 65-year old biological mother reported that at age 58 years her head had turned to the right after minor head trauma, 7 years after the onset of dystonia in her daughter. Neurological examination (Video 2) revealed her head turned to the right, with mild right laterocollis. CD was more severe in certain positions and while walking. Mild dystonic posturing of the arms was observed. Symptoms were relieved with a sensory trick. She did not complain of pain. Also in this case, all neurological investigations were negative. A diagnosis of iCD was made. Treatment with botulinum toxin (Botox) injections (40 U injected in the left SCM, 25 U in the right SCM, and 30 U in the right splenius capitis) led to symptom improvement. Therapy continues with Botox injections every 3 months.

**Video 2:** **The mother.** Segment 1 shows the patient’s 65-year-old mother, with her head turned to the right. The sensory trick improved the neck turning. CD did not change with distractive maneuvers. Mild dystonic posturing of the arms (mainly the hands and fingers) can observed while she keeps her arms outstretched. Neck turning while walking was also evident.

Blood samples were taken from both patients, and DNA was extracted by standard procedures. A panel of more than 50 genes associated with movement disorders and all known dystonia genes, including the newly discovered Gnal, Thap 1, Ano3, was investigated.

## Discussion

The two cases highlight the caveats in differential diagnosis between iCD and fCD. Indeed, both may be remittent [3,4], vary with movement, fluctuate and ameliorate in certain conditions [5,7]. Moreover, few causative genes are known [1], and neurophysiological, neuroimaging-related biological hallmarks for iCD are lacking.

The case of the daughter is particularly difficult, given the clinical history and results of neurological examination. Challenging elements are the acute symptoms onset and the long and recurrent periods of remission. While spontaneous remission has been reported [3,4], it is seldom [4] and cycles of remission and relapse rarely recur more than three times [3]. Similarly, reversal of the direction of head turning, albeit rare, is possible [3]. Therefore, history taking provided few reliable clues. However, inconsistent signs revealed at neurological examination (head turning stopped with distractive maneuvers, mild abasia) strengthened the suspicion of functional overlay. The final diagnosis was supported by the failure of standard treatment for iCD and, above all, by the success of psychological and physical rehabilitation, which led to an improvement in symptoms soon after the first session of treatment. The diagnosis of iCD in the patient’s mother was instead based on consistency in history taking and neurological examination (increased dystonia in certain positions and while walking, response to sensory trick) and response to standard treatment.

The same endophenotypic traits have been reported in both iCD and fCD [8,9,10,11,12]. Abnormal inhibition within the motor system is a neurophysiological marker of organic dystonia [8] but it has also been reported in functional dystonia in affected [9] and non-affected sites [10]. Similarly, abnormal somatosensory temporal discrimination is a shared “endophenotypic” trait [11,12], along with similar traits in emotional processing [12], implying the lack of a trustworthy hallmark of the “organic” form.

In the daughter, the diagnosis was additionally challenging because of mild hypertrophy of the left SCM and right shoulder elevation, which persisted at follow up. However, the neurological examination disclosed clearly inconsistent signs, and the positive response to psychological and physical rehabilitation further supports diagnosis of a functional nature of the clinical manifestation. These signs of inconsistency allow for the hypothesis of a functional overlay in this patient, as reported in other neurological disorders. Her case may be viewed a natural example of how endophenotypic traits can predispose to the development of a certain phenotype, i.e., a dystonic phenotype in the two cases, of both an organic and/or a functional type.

Summarizing, the two cases posed a challenge in differential diagnosis and raised some questions. The hypothesis for common endophenotypic traits between organic and function disorders predisposing to a particular phenotype of disease deserves further investigation. Speculations aside, the take-home message is that accurate history taking and neurological examination are essential because CD lacks a “non-clinical” hallmark. Since fCD is rare and difficult to diagnose [3], an additional clue to reach the diagnosis with relative certainty is by disclosing persistent disappearance of the symptoms with psychological and physical rehabilitation.
